# DNA damage in leukocytes after internal *ex-vivo* irradiation of blood with the α-emitter Ra-223

**DOI:** 10.1038/s41598-018-20364-7

**Published:** 2018-02-02

**Authors:** Sarah Schumann, Uta Eberlein, Razan Muhtadi, Michael Lassmann, Harry Scherthan

**Affiliations:** 1 Department of Nuclear Medicine, University of Würzburg, Oberdürrbacher Str. 6, 97080 Würzburg, Germany; 2 Bundeswehr Institute of Radiobiology affiliated to the University of Ulm, Neuherbergstr. 11, 80937 Munich, Germany

## Abstract

Irradiation with high linear energy transfer α-emitters, like the clinically used Ra-223 dichloride, severely damages cells and induces complex DNA damage including closely spaced double-strand breaks (DSBs). As the hematopoietic system is an organ-at-risk for the treatment, knowledge about Ra-223-induced DNA damage in blood leukocytes is highly desirable. Therefore, 36 blood samples from six healthy volunteers were exposed *ex-vivo* (in solution) to different concentrations of Ra-223. Absorbed doses to the blood were calculated assuming local energy deposition of all α- and β-particles of the decay, ranging from 0 to 142 mGy. γ-H2AX + 53BP1 co-staining and analysis was performed in leukocytes isolated from the irradiated blood samples. For DNA damage quantification, leukocyte samples were screened for occurrence of α-induced DNA damage tracks and small γ-H2AX + 53BP1 DSB foci. This revealed a linear relationship between the frequency of α-induced γ-H2AX damage tracks and the absorbed dose to the blood, while the frequency of small γ-H2AX + 53BP1 DSB foci indicative of β-irradiation was similar to baseline values, being in agreement with a negligible β-contribution (3.7%) to the total absorbed dose to the blood. Our calibration curve will contribute to the biodosimetry of Ra-223-treated patients and early after incorporation of α-emitters.

## Introduction

Worldwide, prostate cancer is the second most common cancer in men and has been diagnosed in 1.1 million men in 2012, which accounts for 15% of all incident cancer cases^1^. The majority of patients dying of prostate cancer display bone metastases and varying symptoms such as pain, pathological fractures, neurological disorders, spinal cord compression, and bone marrow failure, all of which are significantly impairing their quality of life^2,3^.

An optimal therapy is seeking to reduce pain, to improve quality of life, and to prolong survival. Various single or multi-modality therapeutic options are available to treat metastasis-induced bone pain. These include analgesics, hormone therapy, chemotherapy, external beam radiation, bisphosphonates, or α- or β^−^-emitting radiopharmaceuticals such as Ra-223 dichloride, Sm-153 EDTMP, and Sr-89 chloride. Of these the α-emitter Ra-223 dichloride is the first licensed radiopharmaceutical that significantly prolongs life in castration-resistant prostate cancer patients with widespread bone metastatic disease as has been shown in the ALSYMPCA trial^4,5^. In contrast to electrons and photons, α-particles have a short path-length of less than 0.1 mm and a high linear energy transfer (LET) and are capable to induce complex chromosome aberrations^6,7^. Several preclinical studies show a higher efficacy when treating tumours with α-particles as compared to the β-emitter Lu-177^8–10^.

Ra-223 dichloride is administered systemically by intravenous injection, thus exposing not only bone metastases but also normal organs and tissues to ionising radiation^11^. As a calcium analogue, Ra-223 dichloride is mainly taken up and incorporated by the bone^11^. However, there is some activity remaining in the blood, even after a few hours and days after administration leading to further irradiation of the blood^12^.

Since the hematopoietic system is a potential organ-at-risk and the absorbed dose to the blood is often used as a surrogate marker for the absorbed dose to the bone marrow in internal dosimetry^13^, it is of great interest to quantify the radiation-induced DNA damage in blood leukocytes after internal irradiation with the α-emitter Ra-223 and to determine the absorbed dose/DNA damage relationship using the early DSB markers γ-H2AX and 53BP1.

Double-stranded DNA damage is particularly threatening a cell’s survival or may lead through misrepair to increased mutation load and is thus of prime interest for clinical applications of genotoxic compounds and ionising radiation. An early event after ionising radiation-induced DNA double-strand break (DSB) formation is the ATM-dependent phosphorylation of the histone H2 variant H2AX, then called γ-H2AX^14–16^. In addition, the damage sensor 53BP1 is recruited to the chromatin domain surrounding a DSB^17,18^ where it co-localises with γ-H2AX^19^ and influences DNA damage repair pathway choice and contributes to the repair of DSBs in heterochromatin^20,21^. Furthermore, 53BP1 can promote the mobility of damaged chromatin^22^. Consequently, radiation-induced DSBs can be addressed by microscopically visible DNA damage protein foci that display both γ-H2AX + 53BP1 DNA damage markers^13,23,24^. DSB foci disappear by 53BP1 dissociation and γ-H2AX dephosphorylation after DSB repair has been completed^20,25^ with 53BP1 often displaying a reduced residency time relative to γ-H2AX. Most *in-vitro* studies of ionising radiation-induced DSB formation indicate a linear relationship between the number of microscopically visible low LET radiation-induced foci and the absorbed dose after external irradiation^14,15^ and after internal *in-vivo* and *in-vitro* exposure^23,26,27^.

Compared to β- and γ-radiation, high LET radiation, such as α-particles, causes DSBs and complex DNA damage which is more difficult to repair^28,29^. Low LET electrons and photons induce localised focal assemblies of DNA repair factors relating to the generation of mostly simple isolated DSBs. DNA damage induced by α-particles, on the other hand, involves the generation of closely stacking DSBs and DNA modifications along the ionisation tracks that can be visualised in exposed nuclei as long tracks of γ-H2AX-positive chromatin and repair associated proteins when the ionisation tracks in the nuclei lie parallel to the focal plane of the observer^30–32^. It has been shown that the quantification of these tracks can be informative for biological dose estimation^24,33,34^. So far, this method has been used to quantify DNA damage to cultured human blood cells after external α-irradiation^24^ and after radon gas exposure^33^, but not after internal irradiation with the clinically used α-emitter Ra-223.

Therefore, we designed a study to investigate and analyse the DNA damage geometry induced by internal (in solution) exposure to an α-emitter. We quantified α-tracks and DNA damage foci in leukocyte nuclei after internal *ex-vivo* irradiation of peripheral blood with Ra-223 and correlated the results to the absorbed dose to the blood. In order to simulate the effects of a Ra-223 dichloride therapy in close match, we used whole blood samples from healthy volunteers and created a low dose and low dose-rate Ra-223 irradiation setup *ex-vivo*. For the calculation of the absorbed doses, the energy deposition of the emitted α- and β-particles was considered and respective absorbed dose coefficients were determined. By transferring the latter parameters to a previously published calibration setup^23^, we here established a calibration of the γ-H2AX assay with peripheral blood leukocytes after internal *ex-vivo* irradiation with the α-emitter Ra-223.

## Material and Methods

### Ethics statement and informed consent

The research plan was presented to the ethics committee of the Medical Faculty of the University of Würzburg, Germany (Az: 165/14). The ethics committee approved the study by stating that there were no objections to the conduct of the study. All procedures performed in studies involving human participants were in accordance with the ethical standards of the ethics committee of the Medical Faculty of the University of Würzburg and with the principles of the 1964 Declaration of Helsinki and its later amendments or comparable ethical standards. Informed consent was obtained from all individual participants included in the study. The blood was drawn in the Department of Nuclear Medicine of the University Hospital Würzburg by experienced physicians of the department. The samples were anonymised for further processing.

### Specifications of Ra-223 and calculation of the absorbed dose to the blood

**Figure 1:**
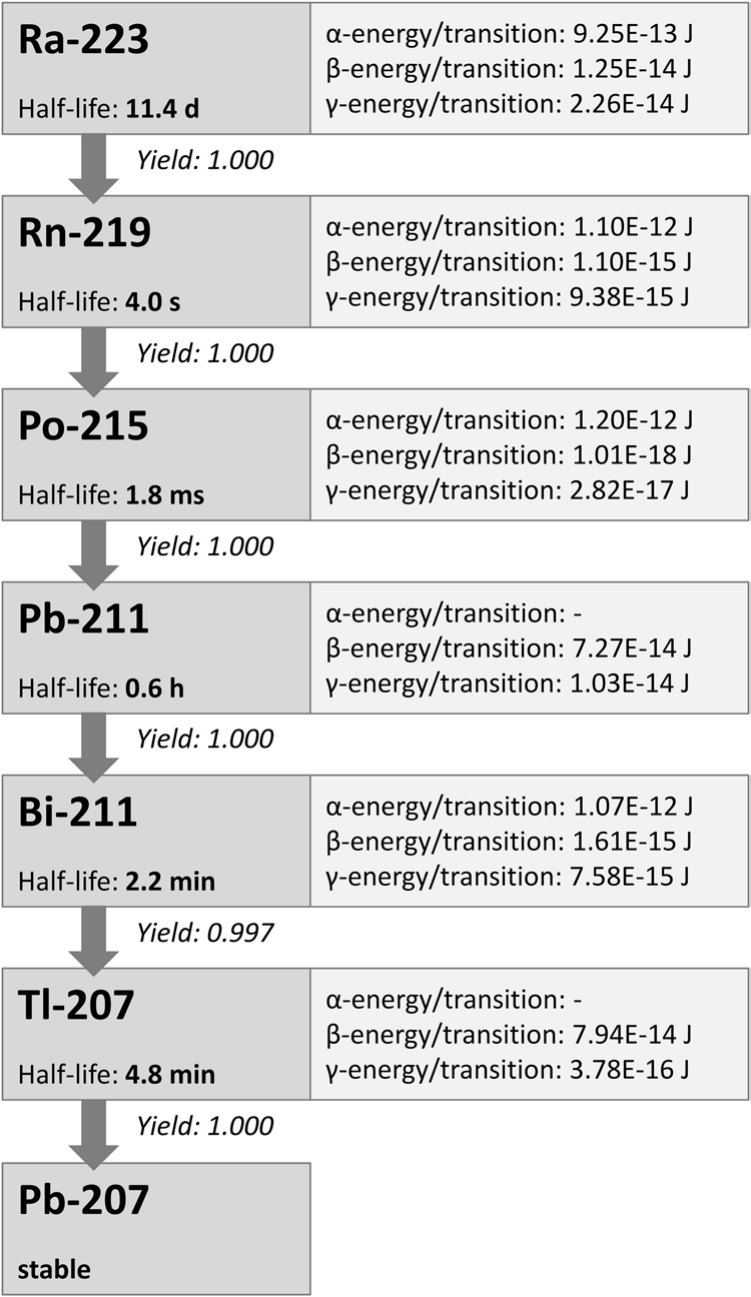
Decay chain of Ra-223 with data on half-lives and energy deposition per transition taken from^35^. The decay of Bi-211 into Po-211 is not shown in this scheme as its yield is less than 0.3%, while its decay into Tl-207 accounts for more than 99.7%.

Ra-223 is an α-emitter with a half-life of 11.43 days. As shown in Fig. 1 it decays in six steps into the stable product Pb-207. Its progeny Rn-219, Po-215 and Bi-211 are also α-emitters so that in total four α-particles are emitted per decay, depositing energies between 5.77 MeV (Ra-223) and 7.49 MeV (Po-215). Here, the decay of Bi-211 into Po-211 is neglected as its yield amounts to less than 0.3%. Considering this branch, an additional α-particle is emitted when Po-211 decays into Pb-207^35,36^.

For the calculation of the absorbed doses to the blood, it was assumed that the energy of all α- and β-particles of all progeny of Ra-223 was deposited locally. The γ-contribution was neglected because of the low interaction probability of the photons in the small volume considered. Based on these assumptions, absorbed dose coefficients (in mGy∙kBq^−1^) after 1 h irradiation were determined for both the α- and the β-contribution to the absorbed dose to the blood.

### Blood sampling, irradiation and isolation of leukocytes

The procedure followed mostly the protocol of Eberlein *et al*.^23^. Briefly, blood samples from six healthy volunteers (3 male and 3 female) aged between 21 and 62 years (Table 1) were drawn using Li-Heparin blood collecting tubes (S-Monovette®; Sarstedt) at different days and were subsequently split into six 3.5 ml aliquots. One non-irradiated blood aliquot of each volunteer was used for detection of the individual background focus rate. Ra-223 dichloride solution (Xofigo®; Bayer) was diluted with phosphate buffered saline (PBS) to result in different activity concentrations. Blood aliquots were then supplemented with 1 ml of the various Ra-223 dichloride solutions containing different activities, followed by incubation at 37 °C for 1 h in round bottom tubes (Sarstedt). To ensure a uniform irradiation of the blood, samples were incubated on a roller-mixer at 35 rpm. For leukocyte separation, 3.5 ml of the Ra-223-containing blood solution were filled into CPT Vacutainer tubes (BD) and centrifuged for 20 min at 1500∙g according to the manufacturer’s instructions. The isolated cells were washed twice in PBS and fixed in a 70% ethanol solution. The fixed leukocytes were stored at −20 °C before they were shipped to the Bundeswehr Institute of Radiobiology in Munich, Germany, where they were stained for γ-H2AX + 53BP1 and analysed.

**Table 1:** Demographic data of the volunteers and their fit parameters based on α-track frequency.

Volunteer ID	Gender	Age (a)	Slope (mGy^−1^)	Intercept
TP 1	m	59	0.191 ± 0.042	0.651 ± 0.863
TP 2	f	62	0.239 ± 0.028	0.327 ± 0.975
TP 3	m	22	0.203 ± 0.034	0.057 ± 0.868
TP 4	m	29	0.242 ± 0.036	1.263 ± 0.924
TP 5	f	21	0.240 ± 0.037	−0.061 ± 0.868
TP 6	f	34	0.205 ± 0.024	−0.398 ± 0.781

The slope and the intercept values relate to the number of α-tracks per 100 cells.

### Activity quantification

To quantify the exact activity concentration in each blood aliquot, up to 1 ml of each sample was measured in a calibrated, high purity germanium detector (Canberra). The counting efficacy of the detector was ascertained by measuring several NIST- and NPL-traceable standards. Three different γ-emission lines of Ra-223 (269.5 keV, emission probability of 14.2%) and its progeny Rn-219 (271.2 keV, 11.1%) and Bi-211 (351.0 keV, 13.0%) were evaluated. All measurements were decay corrected to the start time of the measurement.

### Immunofluorescent staining and evaluation of DNA damage

The ethanol-fixed cells were subjected to cyto-centrifugation followed by immunofluorescent staining to detect DNA damage-associated protein accumulation as microscopic foci at DNA double-strand break sites as described by Ahmed *et al*.^37^. Primary antibodies against γ-H2AX (Mouse anti-γ-H2AX; Merck) and 53BP1 (Rabbit anti-53BP1; Novus) were applied, and detected with secondary goat anti-mouse Alexa-488 (Mobitec) and donkey anti-rabbit Cy3-labeled antibodies (Dianova). The number of radiation-induced DNA damage and repair protein tracks and foci was analysed by the same experienced investigator (HS) in 100 leukocyte nuclei per sample by manual counting directly in a Zeiss Axioimager 2i fluorescence microscope of the ISIS fluorescence imaging system (MetaSystems) equipped with green and red double band pass filters (AHF analysentechnik). Images were recorded at 630× magnification with a Carl Zeiss Plan-Apochromat 63×/1.40 oil lens with a XY resolution of 0.25 µm at 550 nm. Fluorescence intensity profiles were obtained in digital images of the ISIS fluorescence imaging system using the profile option. The diameter of rounded γ-H2AX + 53BP1-containing foci was determined in digital RGB images recorded at the focal plane of the foci using the “length measurement” and “signal normalization” function of the ISIS imaging system (MetaSystems). Cells that showed deformed or overlapping nuclei were excluded from analysis.

**Figure 2:**
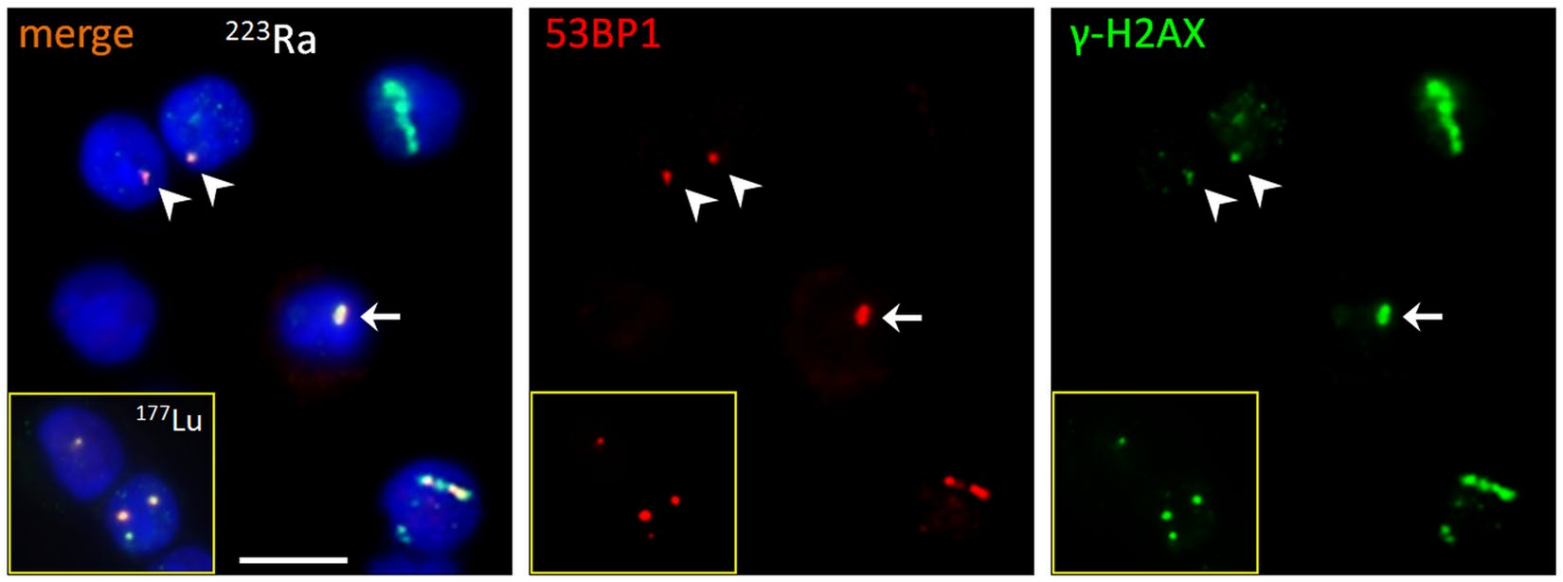
Composite image showing typical nuclei (DAPI, blue) with α-particle tracks (to the right), a large focus (central nucleus, arrow) and nuclei with small foci (arrow heads), the latter likely representing β-induced simple DSBs. Small foci only are seen in nuclei in the yellow-boxed inset derived from a Lu-177 β-exposed blood sample. The red channel shows 53BP1 fluorescence signals, the green channel the γ-H2AX signals only. α-tracks are often disrupted along their length with variable distribution of the two DSB markers used, likely owing to different chromatin densities along the tracks. Rarely, α-tracks failed to display visible 53BP1 accumulations at the track structures (except for a faint background); such tracks usually had a broad γ-H2AX distribution (upper right nucleus) suggesting that they were formed early during exposure. Conversely, tracks with a thinner γ-H2AX distribution always contained 53BP1 (nucleus to the lower right). The central nucleus displays a large (>1.1 µm) α-induced focus (arrow). Magnification bar: 10 µm.

**Figure 3:**
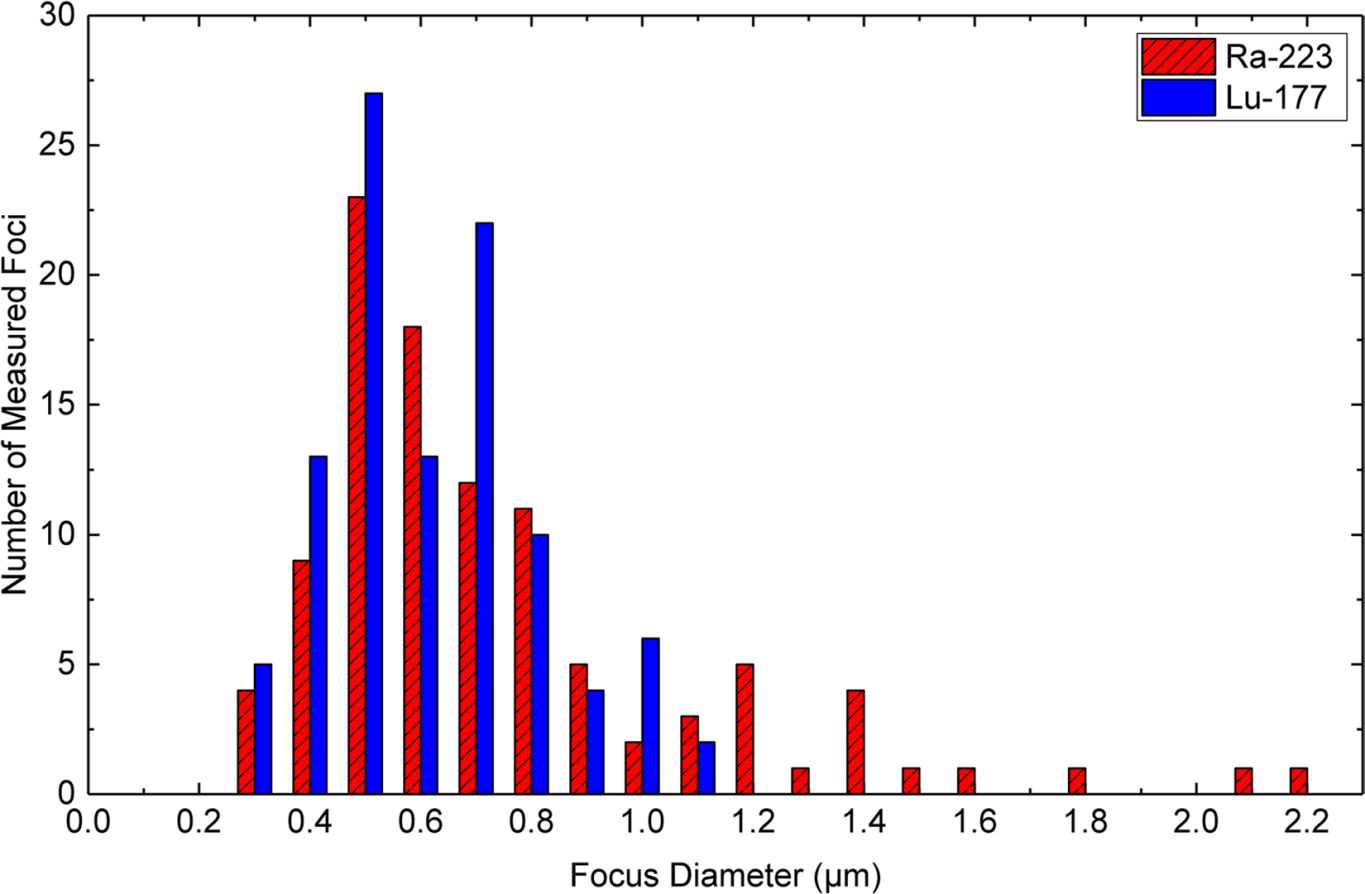
Size distribution of 102 rounded γ-H2AX + 53BP1 foci of Ra-223 (α) or Lu-177 (β^−^, see^23^) irradiated leukocytes (see Fig. 2). Only α-irradiated leukocytes displayed foci larger than 1.1 µm, thus these foci are considered to reflect α-tracks perpendicular to the focal plane.

Based on the measurement of 102 foci per nuclide, DNA damage could be categorised into two classes, I) distinct small round foci typically seen in β-irradiated mononuclear blood cells^23^ (Ø ≤ 1.1 µm diameter; see Figs 2, 3) and II) cells containing α-induced γ-H2AX tracks (Fig. 2) and/or cells with large foci (Ø > 1.1 µm) likely resulting from α-hits laying perpendicular to the observed focal plane (Figs 2, 3).

### Statistical analysis

For data analysis and statistical evaluation Origin (OriginPro 2017, Origin Lab Corporation) was used. To test whether data were distributed normally, the Shapiro-Wilk test was conducted. Results were considered as statistically significant for p < 0.05.

For the average number of α-tracks and small foci per cell the standard deviation of each value was calculated assuming a Poisson-distribution.

For the α-contribution to the absorbed doses to the blood a propagation of uncertainties was performed assuming that the activity, the time between the addition of the radioactive solution and the measurement in the germanium detector, the measuring volume and the absorbed dose coefficient are independent variables with uncertainties.

### Data availability

The datasets generated during this study are available from the corresponding author on appropriate request.

## Results

### Absorbed dose calculation and activity quantification

Considering the energy deposition of all α- and β-particles, an absorbed dose coefficient of 16.1 mGy∙kBq^−1^ was obtained for 1 ml of blood at 1 h irradiation. The contribution of the α-particles to the total absorbed dose to the blood, hereinafter referred to as α-dose, was 96.3% so that an α-dose coefficient of 15.5 mGy∙kBq^−1^ was used for the calculations. Accordingly, an absorbed dose coefficient of 0.6 mGy∙kBq^−1^ was used to calculate the contribution of the β-particles to the total absorbed dose to the blood (β-dose), accounting for 3.7%.

For activity quantification, in average 2.23E + 04 counts were measured for the combined Ra-223 and Rn-219 peak at 270 keV and 0.94E + 04 counts for the Bi-211 peak at 351 keV. There was equilibrium for Ra-223 and its progeny.

The activity concentration in the blood samples ranged between 0.40 kBq∙ml^−1^ and 9.13 kBq∙ml^−1^ at the time when the radioactive solution was added.

### α-track characteristics

**Figure 4:**
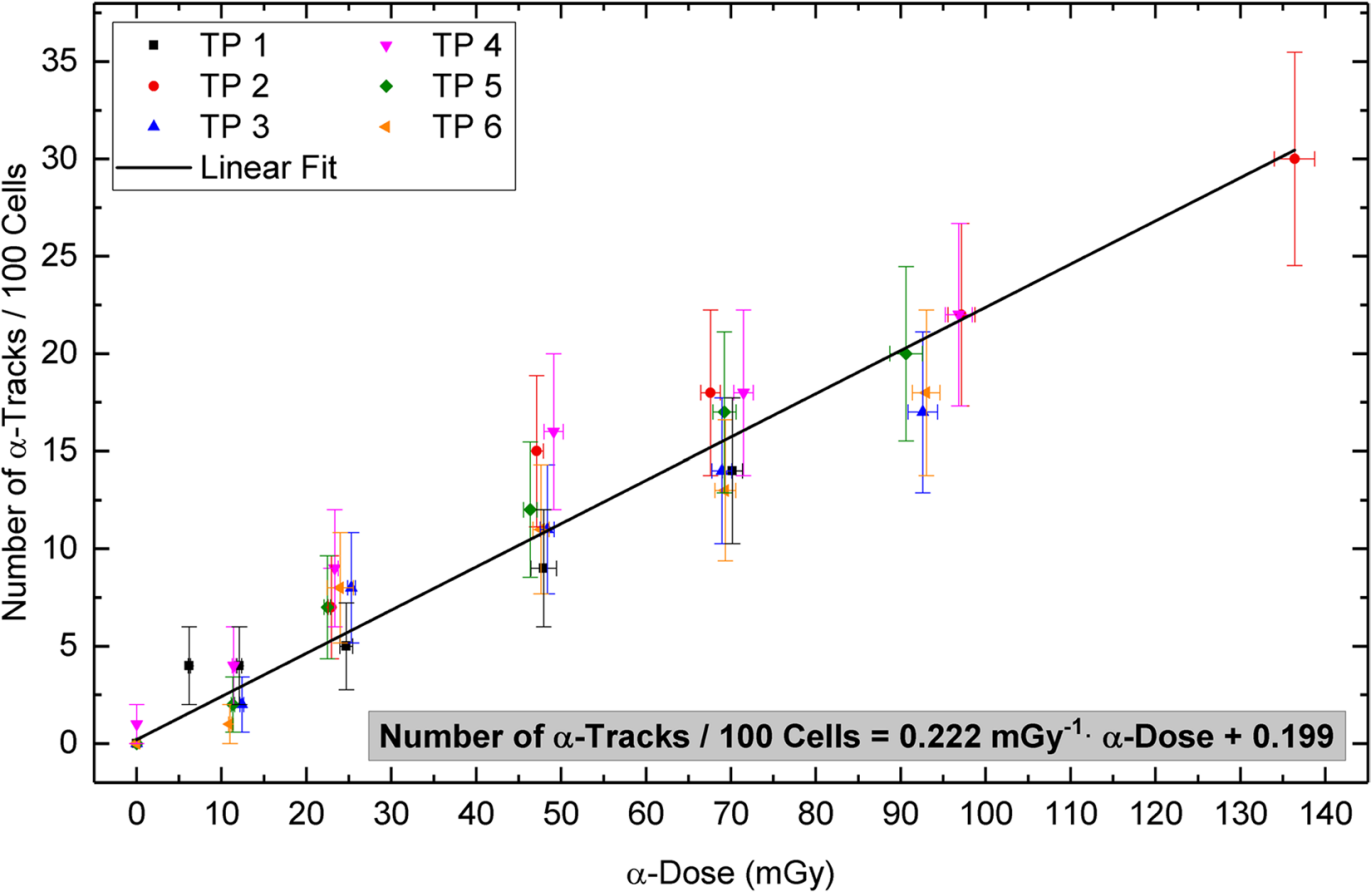
Number of α-tracks in 100 cells as a function of the α-dose with a linear fit to the pooled data of blood samples from six volunteers. The error bars along the X-axis represent the uncertainties of the α-dose calculation. The error bars along the Y-axis represent the standard deviation of each sample assuming a Poisson distribution.

**Figure 5:**
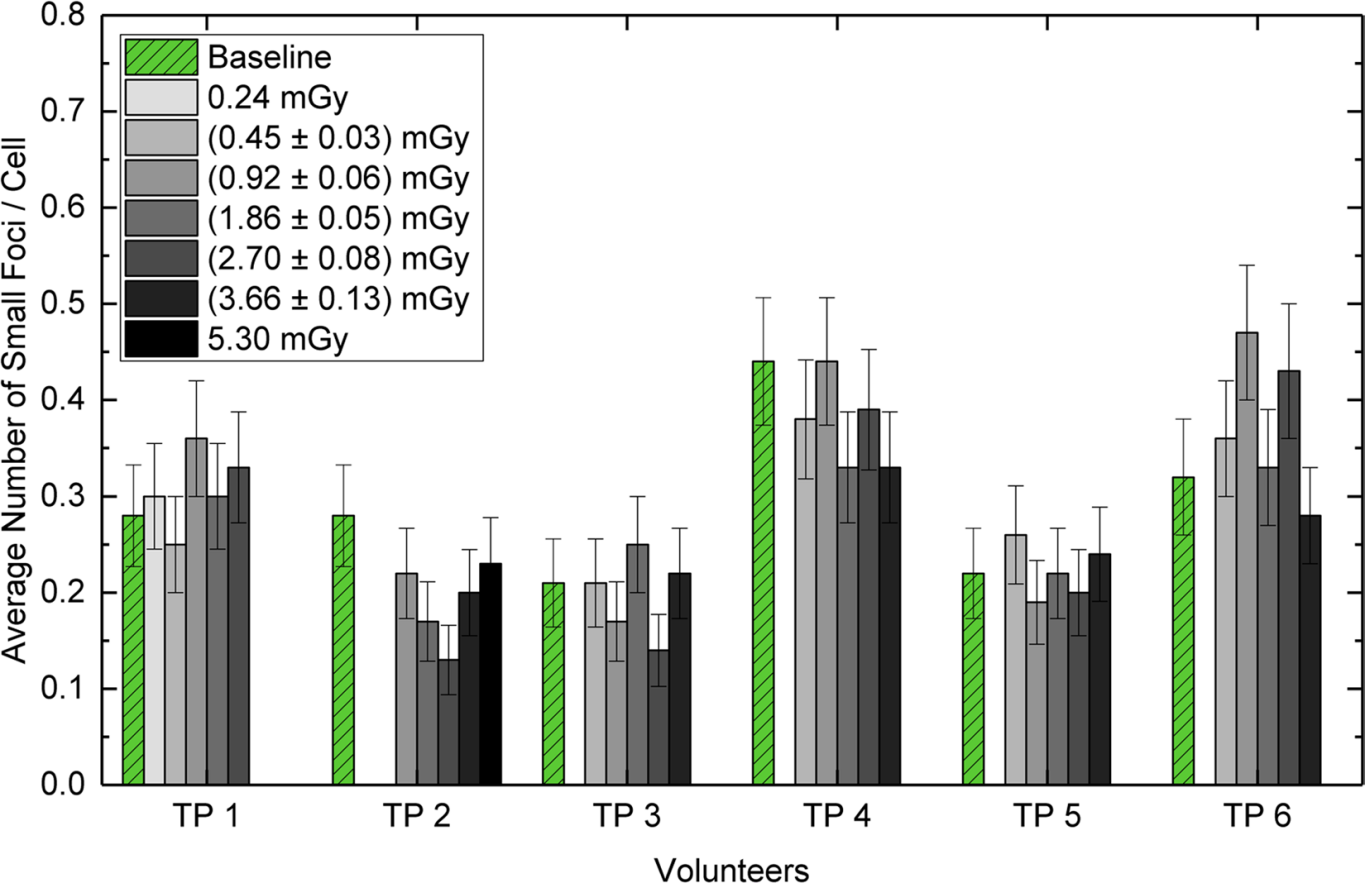
Ra-223 β-dose contribution to the focus count. The bar plot shows the average number of foci per cell in the baseline samples (green, with pattern) and in the five irradiated samples (grey scale) for each volunteer. The grey shaded bars indicate the β-doses as stated in the legend box, denoting the mean values of the individual β-doses with the maximum range. The blank positions for some bars are due to absence of samples with the respective β-doses. The error bars in the plot represent the standard deviation of each foci value assuming a Poisson distribution.

Cells exposed to α-particles display size and morphology differences in the DNA damage geometry, which is a consequence of high LET radiation that delivers densely spaced ionisation events along the particle track. 53BP1 and γ-H2AX form signal tracks consisting of closely stacked DSB sub-compartments, which contrasts with the randomly distributed small foci after low LET irradiation that likely contain simple DSBs^38,39^. Recently, the linear geometry of these α-tracks has proven to be informative in monitoring DNA damage in mononuclear blood cells of rats^34^ and in human peripheral blood lymphocytes^33^ after radon gas exposure. Here, we enumerated the occurrence of cells with linear α-tracks and huge damage foci (Ø > 1.1 µm) as well as the number of small foci in 100 leukocytes per Ra-exposed blood sample (Fig. 2). The huge foci with diameters of 1.2  µm and larger were assumed to be tracks of α-particles having traversed the cells perpendicular to the slide plane as these were not observed in β-exposed leukocytes (Fig. 3). The average track numbers (including the perpendicular tracks) and small foci numbers were plotted individually against the α-doses and β-doses, respectively (Figs 4, 5).

### Dose dependency of the α-tracks

In total, 36 blood samples with α-doses ranging from 0 to 136 mGy were evaluated. The number of α-tracks observed per 100 cells ranged from 0 to 30. In the 600 cells of the non-irradiated baseline samples of six volunteers (Table 1), only one cell with a single α-track was detected. All the irradiated samples contained cells with α-tracks, even at an α-dose of 6.3 mGy. Cells with two α-hits were rarely seen (8/3000 cells) in our setup. This contrasts with widespread γ-H2AX formation in nuclei experiencing more traversing α-particles per cell corresponding to higher α-doses^24,40^.

Taking into account the standard deviation of the number of the α-tracks and the uncertainties of the α-dose calculation, individual linear fits were performed for the data of each volunteer (Table 1). The values of the slopes were normally distributed (p = 0.09) with a mean value (±SD) of (0.220 ± 0.023) mGy^−1^. There was no correlation between the slope values and the age of our volunteers. One-way ANOVA revealed that the population means of the slopes of male and female volunteers were not significantly different.

In order to create a calibration curve, the data points of all volunteers were pooled and a linear fit was performed (Fig. 4). The linear equation of the calibration curve is:

$${\rm{Number}}\,{\rm{of}}\,{\rm{\alpha }} \mbox{-} \mathrm{tracks}/{\rm{100}}\,{\rm{cells}}=0.222\,{{\rm{mGy}}}^{-1}\,\cdot \,\alpha  \mbox{-} {\rm{dose}}+0.199$$

The standard errors of the slope and the intercept are 0.014 mGy^−1^ and 0.354, respectively.

### β-particle-induced damage

Since Ra-223 and its progeny also emit β-particles, we determined the average number of small γ-H2AX + 53BP1 foci in each sample. In our setup, small foci with diameters smaller than 1.2 µm are present in low LET irradiated cells (e.g., Lu-177, Fig. 3). Therefore, such foci, that are assumed to be induced by β-particles, were analysed in addition to α-tracks in all samples. The average small foci number per cell of the baseline samples ranged from 0.21 to 0.44. There was no correlation between the baseline values and the age of our volunteers. In the irradiated samples, the average number of small foci per cell was similar to the corresponding baseline value for each volunteer and did not correlate with the calculated β-dose, which accounted only for 3.7% of the total absorbed dose to the blood and was therefore 5.3 mGy at the maximum for all samples (Fig. 5).

## Discussion

The present study provides a first correlation of the absorbed doses to the blood and the number of radiation-induced γ-H2AX foci tracks after internal *ex-vivo* exposure to the clinically used α-emitter Ra-223. The range for the absorbed doses to the blood (0 to 142 mGy) was chosen to match with our former calibration curve established with the β-emitters Lu-177 and I-131^23^. This dose range was also in accordance with a study on chromosomal aberrations in peripheral lymphocytes of patients treated with Ra-224 for ankylosing spondylitis^41^.

Here, we observed a linear relationship between the frequency of α-induced DNA damage tracks and α-doses after internal irradiation with Ra-223 and its progeny, which enabled us to generate a first *ex-vivo* calibration curve. Measurements also showed that Ra-223 was in equilibrium with its progeny in our samples, thus matching the clinical situation in a patient.

We noted that the frequency of α-tracks represents a suitable parameter for biological dose estimation, which is in accordance with recent γ-H2AX studies on DNA damage after internal or external irradiation with α-particles using the γ-H2AX assay^24,33,34,42^. We observed that γ-H2AX was a more robust marker for α-tracks than 53BP1, in agreement with previous observations that γ-H2AX is an indirect DSB marker in the chromatin surrounding DSBs in high LET particle tracks^38,43,44^. 53BP1 was usually less abundant in the damage tracks of irradiated leukocytes and even absent from a few tracks. The latter agrees with Ding *et al*. who also observed that 40% γ-H2AX tracks in Rn-222 exposed lymphocytes failed to display 53BP1, in their setup, however, after 1–6 hours of radon exposure^33^. Since both 53BP1 and γ-H2AX occur in the chromatin domain surrounding a DSB^45^ and DSB repair proceeds along different routes in eu- and heterochromatin^46,47^, it may be that the failure of 53BP1 to distinctly accumulate at rare γ-H2AX tracks may relate to failure of staining in a particular cell, or chromatin differences, or the fact that pathway decisions involving 53BP1 have already been made and 53BP1 has left the place^20^. This would align with the observation that 53BP1 and γ-H2AX display different retention times at DSBs^48^. Finally, it cannot be excluded that an occasional or rare type of cell may have failed to express 53BP1.

The number of DSBs in a high LET particle trajectory is hard to address experimentally due to the limited spatial resolution of conventional microscopy^49,50^. Consequently, studies with ultra-resolution microscopy will, most likely, reveal more details of the substructures and DSB numbers hidden in γ-H2AX-positive DNA damage trajectories after α-particle irradiation^47,51,52^. At an even higher resolution, quantitative electron microscopy studies suggest the stacking of up to 500 DSBs per µm^3^ in high LET particle-induced tracts of fibroblast nuclei^53^. In future it will thus be interesting to learn about the numbers of DSBs in human lymphocyte nuclei after α-irradiation.

Additionally to the number of α-tracks, we also analysed the number of small foci, indicative of DSBs induced by the low LET β-particles of Ra-223 and its progeny. Small foci in α- and β-exposed leukocytes ranged from 0.3 µm to 1.1 µm in diameter, with the smallest foci being similar to the 53BP1 substructures detected by 4Pi microscopy^49^. However, the number of radiation-induced foci was not elevated relative to the controls, which is most likely due to the low β-doses absorbed (maximally 5.3 mGy).

The treatment of patients with Ra-223 has been simulated by Horn *et al*. who externally irradiated isolated human lymphocytes on top of a 37 kBq Am-241 source^24^. They observed that multiple α-particle traversals of one cell induced a pan-nuclear γ-H2AX staining whose intensity depends on the number of tracks in the cell^24^. Extensively phosphorylated pan-γ-H2AX chromatin was also seen in cancer cells hit by multiple α-particles in the range of up to several Gy^40^. Unfortunately, the study of Horn *et al*. failed to provide values for the absorbed dose to the lymphocytes by α-particle irradiation and consequently, no correlation of the number of tracks and nuclei with pan-γ-H2AX formation to the absorbed doses could be established.

Similar to our present study, Ding *et al*. also observed a linear correlation between the frequency of γ-H2AX tracks and the cumulative absorbed dose in *in-vitro* Rn-222 exposed human blood lymphocytes^33^. Yet, their absorbed doses (1.74–6.85 mGy) and γ-H2AX signal track frequencies were about one magnitude lower and the slope of the linear curve was 55% smaller relative to the slope value we obtained here. With respect to dose estimation it is of note that Ding *et al*. considered cumulative absorbed α-doses determined by a solid-state CR-39 track detector and did not consider any β-contribution. In contrast, we used decay data and activity measurements of each blood sample to calculate α- and β-doses. Ding *et al*. also obtained a linear dose-response relationship for individual γ-H2AX foci frequencies and, in contrast to our observations, they found that the number of γ-H2AX foci was significantly higher than the baseline value even at a cumulative dose of 1.74 mGy. However, the different irradiation conditions and counting criteria make a direct comparison to our results problematic. Ding *et al*. irradiated isolated lymphocytes *in-vitro* with radon gas in an angle range between 0 to 180 degrees for 1–6 h whereas we uniformly distributed the Ra-223 solution in the blood by constant mixing for 1 h. With regard to cell evaluation and track geometry, Ding *et al*. counted only linear γ-H2AX foci tracks and therefore did not consider the tracks of the α-particles having traversed the cells perpendicular to the slide plane. Due to focus frequencies in the per mill range, Ding *et al*. had to enumerate linear γ-H2AX foci tracks and foci in 2000–4000 cells, while our setup revealed track frequencies in the percentage range, which relates to the different absorbed doses monitored. In our setup, evaluation of 100 cells per sample sufficed to reveal an α-exposure, which may be beneficial for biodosimetric applications.

In summary, our study provides a first approach to biological dosimetry of Ra-223-internally irradiated blood samples, closely mimicking the irradiation of patient blood during Ra-223 dichloride therapy. The results presented in this work are a first step towards retrospective biodosimetry of patients after incorporation of an α-emitter. While the data obtained so far are valid for blood withdrawn at around one hour after the intake of the isotope, future *ex-vivo* and *in-vivo* studies will address the time dependency of the dose response. It is expected that this approach will contribute to the development of a quantitative biodosimetry tool with well-defined uncertainties.
